# Harnessing *Cannabis sativa* Oil for Enhanced Skin Wound Healing: The Role of Reactive Oxygen Species Regulation

**DOI:** 10.3390/pharmaceutics16101277

**Published:** 2024-09-30

**Authors:** Dipa K. Israni, Neha R. Raghani, Jhanvi Soni, Mansi Shah, Bhupendra G. Prajapati, Mehul R. Chorawala, Supachoke Mangmool, Sudarshan Singh, Chuda Chittasupho

**Affiliations:** 1 Department of Pharmacology, L J Institute of Pharmacy, L J University, Ahmedabad 382210, Gujarat, India; dr.dipa.israni@ljku.edu.in (D.K.I.); mansi.shah_ljip@ljinstitutes.edu.in (M.S.); 2 Department of Pharmacology and Pharmacy Practice, Saraswati Institute of Pharmaceutical Sciences, Gandhinagar 382355, Gujarat, India; rrneha2910@gmail.com; 3 Department of Pharmacology, Parul Institute of Pharmacy, Parul University, Waghodia, Vadodara 391760, Gujarat, India; sonijhanvi4@gmail.com; 4 Shree S. K. Patel College of Pharmaceutical Education and Research, Ganpat University, Kherva 384012, Gujarat, India; bhupen27@gmail.com; 5 Faculty of Pharmacy, Silpakorn University, Nakhon Pathom 73000, Thailand; 6 Department of Pharmacology and Pharmacy Practice, L. M. College of Pharmacy, Opp. Gujarat University, Navrangpura, Ahmedabad 380009, Gujarat, India; mehulchorawala@gmail.com; 7 Faculty of Pharmacy, Chiang Mai University, Chiang Mai 50200, Thailand; supachoke.man@cmu.ac.th; 8 Office of Research Administration, Chiang Mai University, Chiang Mai 50200, Thailand

**Keywords:** antioxidant, cannabis oil, *Cannabis sativa*, reactive oxygen species, skin wound healing

## Abstract

*Cannabis sativa* emerges as a noteworthy candidate for its medicinal potential, particularly in wound healing. This review article explores the efficacy of cannabis oil in reducing reactive oxygen species (ROS) during the healing of acute and chronic wounds, comparing it to the standard treatments. ROS, produced from various internal and external sources, play a crucial role in wound development by causing cell and tissue damage. Understanding the role of ROS on skin wounds is essential, as they act both as signaling molecules and contributors to oxidative damage. Cannabis oil, recognized for its antioxidant properties, may help mitigate oxidative damage by scavenging ROS and upregulating antioxidative mechanisms, potentially enhancing wound healing. This review emphasizes ongoing research and the future potential of cannabis oil in dermatological treatments, highlighted through clinical studies and patent updates. Despite its promising benefits, optimizing cannabis oil formulations for therapeutic applications remains a challenge, underscoring the need for further research to realize its medicinal capabilities in wounds.

## 1. Introduction

Wound healing is a natural biological process that includes vasoconstriction and coagulation, inflammation, cellular proliferation, and wound remodeling [1,2,3,4,5]. Oxidative stress (OS) plays a crucial role in regulating wound healing. Research over the past decade shows that OS can have both positive and negative effects on wound healing, depending on its context. In mammals, OS arises from either an overproduction of ROS or a reduction in antioxidant defense mechanisms [6]. Wound healing involves inflammation, angiogenesis, and the formation of granulation tissue. These processes are closely linked to upregulations in OS and ROS levels [7]. ROS are crucial in all the stages of wound healing. Low levels of ROS are essential for combating invading microbes and activating cell survival signaling [8].

Excessive and uncontrolled oxidative stress is a key factor in both sustaining and disrupting inflammatory processes, which are critical in the development of chronic non-healing wounds [9]. ROS include highly reactive substances such as superoxide anion (O^2•−^), hydrogen peroxide (H_2_O_2_), and the hydroxyl radical (^•^OH). Under normal physiological conditions, mild to moderate levels of ROS play a role in cellular signaling and immune responses. However, external events like injury, inflammation, or radiation can dramatically increase ROS production. This surge leads to oxidative stress within cells, causing damage to skin tissues [10]. The excessive formation and accumulation of ROS beyond the cellular capacity to neutralize them can impede the transition of injured tissue from the inflammatory phase to the proliferative phase of wound healing [11]. As a result, prolonged inflammation in the wound area disrupts the healing process of skin. Maintaining a balance in the redox (reduction–oxidation) within cells can help prevent abnormal cell growth and immune dysregulation. Scientific studies have demonstrated that antioxidants can speed up wound recovery, particularly in cases of chronic wounds [12].

Consequently, using antioxidant components has emerged as an effective strategy to accelerate the healing of chronic wounds. The relationship between ROS and skin injuries has been extensively studied, spurring interest in materials that eliminate ROS for skin wound recovery. Research suggested that cannabidiol (CBD) and cannabis-based therapies may protect against oxidative stress, potentially reducing inflammation and cell damage in the chronic wound microenvironment and the surrounding tissues [13,14]. Based on a comprehensive review, a search has been done for the words CBD, *Cannabis sativa* oil, hemp oil, and reactive oxygen species in various databases, such as PubMed, Science Direct, Google Scholar, and Cochrane library. Patents for the past ten years and those articles which were found to be relevant and have up-to-date information regarding the protective effects of cannabis oil on skin wound healing and a regulatory impact on ROS as well as its molecular mechanism have been selected for inclusion.

*Cannabis sativa* L. (also known as hemp) is an annual plant, part of the Cannabaceae family, that thrives in various habitats [15]. Numerous scientific studies support the therapeutic properties of cannabis, including its anti-inflammatory [16,17,18], antidiabetic [19], neuroprotective [20], anticancer [21,22], antioxidant [23], antimicrobial [24,25], antiviral [26], and antifungal [27] effects. It is used to treat a wide range of conditions, such as skin disorders, cancer, Alzheimer’s disease, Parkinson’s disease, epilepsy, and post-traumatic stress disorder. However, it can also cause adverse effects, like a loss of appetite, chronic pain, and nausea [28]. The medicinal effects of cannabis are influenced by various phytochemicals, which differ in their quantity, stability, volatility, pharmacological activity, physicochemical characteristics, and combinations [29].

Cannabidiol is the non-psychoactive compound found in the *C. sativa* (L.) plant, known for its significant anti-inflammatory properties and its ability to promote wound healing [30,31,32]. Cannabis oil is rich in carotenoids, including flavonoids, terpenes, chlorophylls, and phytosterols, and is a source of β-carotene, lutein, and zeaxanthin [18,33]. These carotenoids have antioxidant properties that scavenge free radicals and protect against UV light [34]. They also enhance skin moisturization, aid in wound healing, and stimulate the synthesis of collagen and elastin by activating fibroblasts. β-carotene can inhibit the activation of pro-inflammatory cytokines caused by ultraviolet B (UV-B) light, demonstrating anti-inflammatory properties. Chlorophyll, a naturally occurring compound in cannabis oil, stimulates tissue development and has antimicrobial qualities, making it effective in treating wounds and skin conditions such as acne, eczema, and ulcers. Chlorophyll is also responsible for the green color of hemp seed oil [15].

The presence of flavonoids, terpenes, carotenoids, chlorophylls, and phytosterols in cannabis seed oil contributes to its anti-inflammatory and anti-aging effects. The oil has a high absorption rate and does not clog pores, making it valuable for skin-soothing formulations including sunscreen, creams, and lotions. Topical lotions and ointments based on cannabis oil show promise in anti-aging skincare [15].

Research on wound-healing agents is a rapidly expanding area in biomedical science. Early findings suggest that bioactive compounds e.g., asiatic acid, quercetin, curcumin, myricetin, calophyllolide, lupeol, catechin, gallic acid, resveratrol, and naringenin may offer significant benefits by enhancing various aspects of the wound-healing process [35]. Several plants have been professionally examined to evaluate their wound-healing activities in various in vivo models and in human beings [36], including *Aloe vera* [37], *Curcuma longa* [38,39,40,41], *Achillea millefolium* [42,43], *Azadirachta indica* [44,45], *Althaea officinalis* [46], *Calendula officinalis* [47], *Camellia sinensis* [48,49], *Eucalyptus camaldulensis* [50], *Hibiscus sabdariffa* [51], *Matricaria chamomilla* [52], *Punica granatum* [53], *Mimosa pudica* [54], *Ocimum gratissimum* [55], *Psidium guajava* [56], and *Simmondsia chinensis* [57,58].

## 2. The Skin’s Structure and Function, and the Pathogenesis of Wounds within the Healing Process

The skin is the largest organ of the human body, serving multiple essential functions [59], such as protection from mechanical injury, pathogens, and harmful substances, while also reducing water loss. Further, the presence of numerous nerve endings help with touch, temperature, pain, and pressure sensations. Similarly, the skin helps to regulate body temperature through the production of sweat and the dilation or constriction of blood vessels. Moreover, the presence of immune cells, such as Langerhans cells, significantly supports the detection of and response to infections. Additionally, in the presence of sunlight, the skin synthesizes vitamin D, which is crucial for calcium and phosphorous absorption and ultimately the maintenance of bone health [1].

A wound occurs when the epidermis, the tissue of an organ, or the mucosal surface is physically disrupted, deviating from its natural physiological structure. This disruption can result from various causes, including illness, accidents, or other factors [36]. Wounds arise when harmful elements compromise the integrity of normal skin tissue. If the healing process is inadequate, wounds can impair the skin’s normal functioning and weaken the body’s immune system [8].

**Figure 1:**
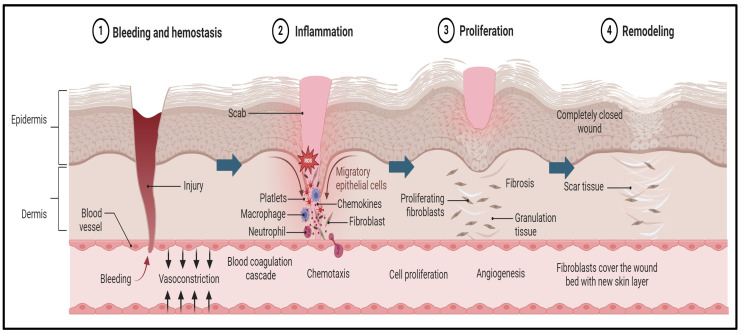
Pathogenesis of wound and the wound-healing process. The first stage begins immediately after tissue injury, with an aim to stop the bleeding and promote blood clot formation. This process is further supported by platelets and clotting factors that generate a provisional matrix that acts as a scaffold for the subsequent stages of healing. Later, the inflammation stage begins, which is often associated with a negative connotation. The inflammation process supports the removal of debris, the clearing of pathogens, and the initiation of the recruitment of various cells, such as neutrophiles and macrophages, that release and regulate the supporting growth factors. Then, cytokines stimulate the subsequent phase of healing, known as proliferation, in which new tissue forms, driven by fibroblasts and keratinocytes. The fibroblasts produce collagen and the keratinocytes contribute to re-epithelization. The final stage of wound healing is maturation. In this stage, the newly developed tissue undergoes remodeling and maturation to attain its optimal strength and functionality. Subsequently, the collagen fibers reorganize and align along the lines of tension, making the healed tissue more resistant to stress, and excessive scar tissue deposition is minimized through a balance of collagen synthesis and degradation.

Wounds may become chronic due to several factors, such as local infection, malnutrition, autoimmune diseases, diabetes mellitus, or inadequate early wound care [60]. Wound healing is a biomedical process that unfolds in three distinct phases: inflammation, proliferation, and remodeling, as illustrated in Figure 1 [61].

During the inflammatory phase, a plug of platelets and blood clots forms at the wound site. Inflammatory cells migrate to the area and are activated by the complement system. These cells produce essential inflammatory chemicals, proteinases, and the growth factors necessary for tissue formation [50]. Following this, keratinocytes near the wound’s edge migrate and change their phenotype to increase cell division, initiating the healing process and the regrowth of the epithelial layer [56]. Effective wound healing requires a delicate balance among cytokines, growth hormones, extracellular matrix components, and various cell types that work together to restore the skin’s outer layer and structural integrity [51].

Several underlying factors can hinder progress through the stages of wound healing, including chronic illness, diabetes, an inadequate blood flow, narrowed blood vessels, poor nutrition, and aging. Vasoconstriction and the disruption of microcirculation reduce the blood flow and oxygen levels, worsening hypoxia at the wound site and impairing tissue recovery [62]. Chronic wounds may also be influenced by local factors, such as illness, oxygen deprivation, inflammation, and stress. Critical wounds often show persistent, severe inflammation with an excessive presence of neutrophils [63]. Persistent inflammation is associated with the degradation of growth factors and the extracellular matrix, elevated levels of pro-inflammatory cytokines, and the excessive activity of matrix metalloproteinases (MMPs) [64]. An imbalance between the expression of MMPs and the tissue inhibitors of metalloproteinases (TIMPs) leads to tissue destruction outpacing tissue formation and the breakdown of growth factors, ultimately impeding the healing process [65].

Inflammation generates many substances that increase the sensitivity of nociceptors and reduce their tolerance to nociceptive signals. CD11b+ myeloid cells, also known as macrophages, are thought to play a key role in pain sensitivity in incisional injury models [66]. The activation of MAPK pathways by calcitonin gene-related peptides enhances keratinocyte proliferation and triggers NLRP1/caspase−1 inflammasome signaling, which mediates mechanical hyperalgesia, dependent on IL−1β [67].

High and persistent levels of inflammatory mediators, which lead to inflammation, can contribute to the chronic pain associated with chronic wounds. In a chronic wound environment, inflammatory cells produce ROS, and high levels of ROS can damage the structural components of the extracellular matrix and cell membranes, leading to early cell aging (cell senescence) [8]. The increased production of inflammatory mediators (e.g., PGE_2_, TNF-a, IL−6) is linked to various chronic pain conditions, causing damage to nerve fibers and altering central signaling pathways [68]. Fibroblasts from chronic, non-healing wounds often exhibit atypical characteristics, such as a reduced cell division capacity, premature cellular aging, and altered cytokine release patterns. These abnormalities are directly related to the wound’s inability to heal properly [69].

Chronic wounds show impaired healing after injury or rupture, leading to incomplete recovery through various stages. The fluids from chronic wounds can inhibit the proliferation of keratinocytes and fibroblasts, resulting in cellular senescence. This phenomenon is influenced by complex factors, including aging, pathological conditions, and cell injury, all of which impact the healing process [69].

Acute wound healing is marked by a typical inflammatory response with a lower production of inflammatory markers and MMPs. These wounds often result from significant tissue damage, medical procedures, or minor thermal injuries [70]. Fibroblasts with high mitotic capacities contribute to the efficient treatment of acute trauma. The goals of wound care are to prevent further damage, achieve rapid wound closure, restore tissue functionality, help the patient resume normal activities, and enhance the overall quality of life [71]. Complex wounds, which involve both infection and tissue loss, may benefit from personalized therapy approaches [72]. However, post-traumatic infections can exacerbate and enlarge the affected area, posing an ongoing risk to wound healing. The susceptibility to wound infection depends on the type and location of the wound [8].

## 3. Reactive Oxygen Species

Reactive oxygen species are free radicals and reactive molecules that are generated from molecular oxygen through redox reactions or electrical stimulation. Major ROS signaling agents, such as superoxide (O^2•−^) and hydrogen peroxide (H_2_O_2_), are produced by the mitochondrial electron transport chain. Other sources of ROS include endothelial cells, xanthine oxidases, cytochrome P450 (CYP) enzymes, neutrophils, monocytes, cardiomyocytes, lipoxygenases, and nitric oxide synthases [73]. ROS can damage normal cells through various reduction processes, making detoxification essential for cellular survival. In an oxygen-rich environment, cells rely on defense mechanisms to balance ROS production and elimination. When ROS production exceeds the capacity of detoxification and repair mechanisms, OS occurs [74,75].

### Sources of Reactive Oxygen Species

An important exogenous source of ROS is exposure to radiation, both ionizing and non-ionizing. Ionizing radiation, such as gamma rays, generates various radical and non-radical intermediates. This includes the ionization of intracellular water, producing aqueous electrons, hydroxyl radicals (OH•), and H_2_O_2_. Non-ionizing radiation, such as UV-light, can also generate ROS. Specifically, UV-C (<290 nm), UV-B (290–320 nm), and UV-A (320–400 nm) can indirectly produce ROS, like superoxide anion (O_2_^−^), H_2_O_2_, and OH• [76].

Air pollutants, such as cigarette smoke and industrial pollutants (including various nitrogen oxides), are significant sources of ROS that can harm organisms through skin contact or inhalation. Toxins, pesticides, herbicides (like paraquat), and chemicals (such as mustard gas and alcohol) can also trigger oxidative stress by generating ROS during their metabolism within living organisms. This oxidative stress can damage various tissues and organs. Additionally, diseases, bacteria, and viruses can produce ROS either directly or as part of an endogenous response triggered by phagocytes and neutrophils.

Chemotherapeutic agents, such as doxorubicin and daunorubicin, are significant sources of ROS. Additionally, drugs like nitroglycerin, which donate nitric oxide (NO) to the body, can produce ROS. Narcotic medicines and anesthetic gases are also known to generate ROS. A major source of ROS is the high consumption of red meat and ethanol. The iron content in red meat catalyzes ROS formation during digestion, while excessive ethanol consumption damages the mucosal layer, leading to severe oxidative stress [77].

During the initial inflammatory phase of wound healing, activated/recruited neutrophils and macrophages produce ROS as part of the body’s defense mechanism against invading pathogens. While ROS play a crucial role in signaling and defense, excessive ROS can lead to oxidative stress, damaging cellular components and hindering the healing process. Balancing ROS production is essential for optimal wound healing, making antioxidant therapies potentially beneficial in wound care [78].

The primary product of the electron transport complexes I (CI) and III (CIII) in the mitochondrial respiratory chain is superoxide, which is converted to H_2_O_2_ by superoxide dismutase (SOD). Catalase or glutathione peroxidase then further reduces H_2_O_2_ to water [79]. Other significant sources of ROS in cells are NADPH oxidases (NOX), including NOX1–NOX5 and Duox1 and Duox2. These NOX enzymes reduce oxygen to superoxide, a process that requires NADPH. Additionally, ROS formation is enhanced by inflammatory cytokines and growth factors such as tumor necrosis factor-alpha (TNF-α), epidermal growth factor (EGF), and interleukin−1 beta (IL−1β) [80].

Additionally, ROS can be produced by xanthine oxidase, an enzyme involved in the conversion of hypoxanthine to xanthine and then to uric acid. During this oxidation process, molecular oxygen is used, resulting in the production of superoxide and other ROS. Xanthine oxidase can also generate nitric oxide, suggesting a dual role in cell signaling. Furthermore, peroxidases, which are enzymes involved in various oxidative reactions, are another potential source of ROS [81]. Other factors contributing to ROS generation include disease conditions such as circadian rhythm disorders, intestinal insomnia, and neurodegenerative disorders. Additionally, processes like respiratory bursts, ischemia, and related conditions also play a role in ROS production [82].

Other enzymes, besides those generating ROS, can contribute to oxidative stress in wounds. One notable group is MMPs. These enzymes degrade the extracellular matrix components, which is a normal part of wound remodeling. However, in chronic wounds, MMP activity can become dysregulated, leading to excessive tissue breakdown and prolonged inflammation. Together, these enzymes exacerbate the oxidative environment, impeding the healing process and making antioxidant therapies a valuable consideration in wound management [83,84].

## 4. Role of ROS in the Wound-Healing Process

Oxygen is essential for wound sterilization and provides energy for the healing process. Oxygen-dependent redox signaling plays a crucial role in wound repair, with H_2_O_2_ and superoxide serving as intracellular messengers throughout the healing process [85,86,87,88]. Optimal levels of ROS and redox signaling are vital for effective wound healing. ROS reduce blood flow in the immediate area, promote the recruitment of platelets and inflammatory cells, and facilitate the destruction of microorganisms at the injury site. Neutrophils and macrophages produce significant amounts of superoxide and H_2_O_2_ via nicotinamide adenine dinucleotide phosphate (NADPH) oxidase, which is crucial for bacterial destruction and infection prevention [89].

ROS also release signals that stimulate wound healing, including tumor necrosis factor-α (TNF-α) and platelet-derived growth factor [90]. In the proliferation phase, redox signaling promotes fibroblast growth and movement, mediates TGF-β1 signaling for collagen, fibronectin, and basic fibroblast growth factor production, and stimulates angiogenesis through VEGF expression, which encourages endothelial cell division and migration [91]

During the clotting phase of wound healing, ROS production is associated with NADPH oxidases (NOXs) in vascular cells, which are activated by tissue factors that are released from platelets. ROS generation is crucial during inflammation, enabling neutrophils and macrophages to destroy microorganisms through oxidative bursts [89,92]. After wound closure, the ROS produced by NOXs regulate the removal of cells and the remodeling of the extracellular matrix (ECM), contributing to scar maturation, typically occurring 8 to 10 days post-closure. Efficient wound healing depends on a delicate balance between ROS production and elimination [9].

The major signaling pathways in the complex process of wound healing involve a coordinated sequence of cellular events and molecular interactions. The primary pathways include the inflammatory response, the proliferative phase, and the remodeling phase, each regulated by specific signaling cascades [93]. In the initial inflammatory phase, the nuclear factor kappa-light-chain-enhancer of activated B cells (NF-κB) pathway is critical. Upon tissue injury, pro-inflammatory cytokines such as TNF-α and IL−1β activate the NF-κB pathway, which translocates them to the nucleus and induces the expression of various inflammatory genes. This response is essential for recruiting immune cells like neutrophils and macrophages to the wound site, which engulf debris and pathogens, setting the stage for subsequent healing phases [94].

The proliferative phase involves the epidermal growth factor (EGF) and platelet-derived growth factor (PDGF) signaling pathways. EGF binds to its receptor (EGFR) on the surface of keratinocytes and fibroblasts, activating downstream signaling via the MAPK/ERK pathway. This promotes cell proliferation and migration, critical for re-epithelialization and granulation tissue formation. Similarly, PDGF stimulates fibroblasts to produce extracellular matrix components, aiding tissue reconstruction. Transforming growth factor-beta (TGF-β) signaling is paramount in both the proliferative and remodeling phases. TGF-β, through its receptors, activates the Suppressor of Mothers against the decapentaplegic (SMAD) pathway, which regulates the gene expression involved in cell proliferation, differentiation, and extracellular matrix production. This pathway ensures proper wound closure and tissue integrity. Moreover, the vascular endothelial growth factor (VEGF) pathway is essential for angiogenesis, the formation of new blood vessels from existing ones. VEGF binds to its receptor, VEGFR, on endothelial cells, activating the PI3K/AKT pathway, which promotes cell survival, proliferation, and migration, essential for restoring oxygen and nutrients to the healing tissue [95]. Dysregulation in these pathways can lead to chronic wounds or excessive scarring. Thus, understanding and modulating these signaling pathways can enhance therapeutic strategies for improved wound healing outcomes.

## 5. The Application of Cannabis Oil in Wound Healing through ROS Regulation

The treatment of skin wounds is a complex field that combines traditional methods with innovative regenerative strategies. While conventional approaches remain vital, emerging therapies such as stem cell treatments, advanced dressings, and tissue engineering hold great promise for improving healing outcomes, particularly in chronic and non-healing wounds. The therapeutic standard care treatments for wound healing typically include antiseptics and disinfectants and various types of wound dressings [96]. These treatments function by providing antimicrobial activity, reducing inflammation, and providing a protective barrier to shield the wound from further contamination [97]. For chronic wounds, additional advanced treatments like debridement, negative pressure wound therapy (NPWT), and hyperbaric oxygen therapy (HBOT) are often employed. These interventions work together to prevent infection, manage exudate, and promote an optimal healing environment [98]. However, a common limitation of these treatments is their lack of significant antioxidant properties. Reactive oxygen species play a critical role in wound pathophysiology, and addressing this component can enhance the overall healing process [84]. This is where cannabis oil, known for its antioxidant capabilities by scavenging ROS, emerges as a promising adjunct or alternative treatment in wound care.

Cannabis oil stands out in the realm of wound care due to its unique combination of antimicrobial, anti-inflammatory, and antioxidant properties [16].The traditional antiseptics and disinfectants, while effective at eliminating pathogens, can sometimes cause tissue irritation and do not address the oxidative stress caused by ROS. In contrast, cannabis oil provides a gentler antimicrobial effect without the harsh side effects, while also mitigating the oxidative damage caused by ROS through its antioxidant properties. Various antibiotics are crucial for treating bacterial infections in wounds but carry the risk of resistance and allergic reactions. Cannabis oil, with its natural antimicrobial components like CBD and THC, offers an alternative that reduces these risks while also delivering anti-inflammatory benefits [99]. Additionally, anti-inflammatory medications, such as NSAIDs and corticosteroids, effectively reduce inflammation and pain but can have systemic side effects with prolonged use. Cannabis oil offers a holistic approach to managing inflammation, minimizing systemic exposure, and promoting overall skin health and regeneration. Wound dressings are essential for maintaining a moist environment and protecting the wound; however, they can benefit from the addition of cannabis oil, which can enhance their effectiveness by providing added anti-inflammatory and antimicrobial support. In chronic wound care, debridement is necessary to remove necrotic tissue but can be painful and requires skilled personnel [100]. While cannabis oil is not a direct substitute for debridement, its anti-inflammatory properties can reduce the frequency and severity of debridement sessions. Advanced dressings and NPWT are highly effective, but costly and complex. Cannabis oil can serve as a complementary treatment, enhancing the efficacy of these advanced therapies by addressing oxidative stress and inflammation. Similarly, while HBOT is a potent treatment for promoting oxygenation in hypoxic tissues due to wound infections, cannabis oil offers a more accessible and cost-effective alternative with its antioxidant benefits, decreasing the oxidative stress caused by the injury [101,102]. Briefly, while standard wound care treatments are effective in their own right, incorporating cannabis oil can address some of their limitations, particularly in managing ROS and inflammation. This makes cannabis oil a valuable adjunctive treatment in both acute and chronic wound care settings, potentially improving healing outcomes and patient comfort. A GC-MS study performed by Sotto and co-worker and Bakali et. al. demonstrated the presence of major components in *C. sativa* L. oil, including γ-caryophllene (~35%), β-caryophyllene oxide (~19%), α-caryophyllene (~11%), α-pinene (~3%), β-selinene (~3%), α-humulene epoxide II (~3%), 1,8-cineole (~2%), α-sabinene (~1%), β-myrcene (~1%), trans-α-bergamotene (~1%), *allo*-aromadendrene (~1%), γ-gurjunene (~35%), α-selinene (~1%), α-cadinene (~1%), and selina−3,7,(11)-diene [103,104]. The endocannabinoid system (ECS) plays a crucial role in maintaining homeostasis by modulating polyunsaturated omega fatty acid (PUFA) signaling, and whole food-based diets that are rich in PUFAs and full-spectrum hemp oils are increasingly used to support ECS function and overall health. Cannabinoids, like THC and CBD, interact with various receptors and influence PUFA metabolism, affecting inflammation and oxidative stress [105]. Interest in cannabis oil has urged growing research into its potential therapeutic benefits, especially in wound care management. This interest stems from the oil’s key bioactive compounds, such as cannabinoids, terpenes, and flavonoids, known for their anti-inflammatory, antioxidant, and antimicrobial properties. Recent studies highlight the innovative use of cannabis oil in combination with various biomaterials for wound healing. Table 1 summarizes the chemical constituents present in the *C. sativa* (L.) plant and their activities.

Biomaterials such as chitosan and alginate, often used in hydrogels, offer controlled release systems for cannabis oil’s active compounds. Chelminiak-Dudkiewicz et al. investigated chitosan films with varying cannabis oil concentrations [116]. Atomic force microscopy revealed that a higher oil content increased the film’s roughness, enhancing protein adsorption, particularly from human albumin. Although a higher oil content improved the mechanical properties and hydrophilicity, it reduced water vapor permeability, a crucial feature for wound dressings. Toxicity tests indicated that a higher oil content enhanced the antimicrobial effects against *Aliivibrio fischeri*, while cell culture studies confirmed the films’ biocompatibility, suggesting their suitability as wound dressings. These findings demonstrate the potential of combining cannabis oil with biomaterials to leverage both their synergistic effects and inherent properties like biocompatibility and moisture retention, which can enhance wound healing by accelerating re-epithelialization, reducing the infection risk, and improving tissue regeneration.

**Figure 2:**
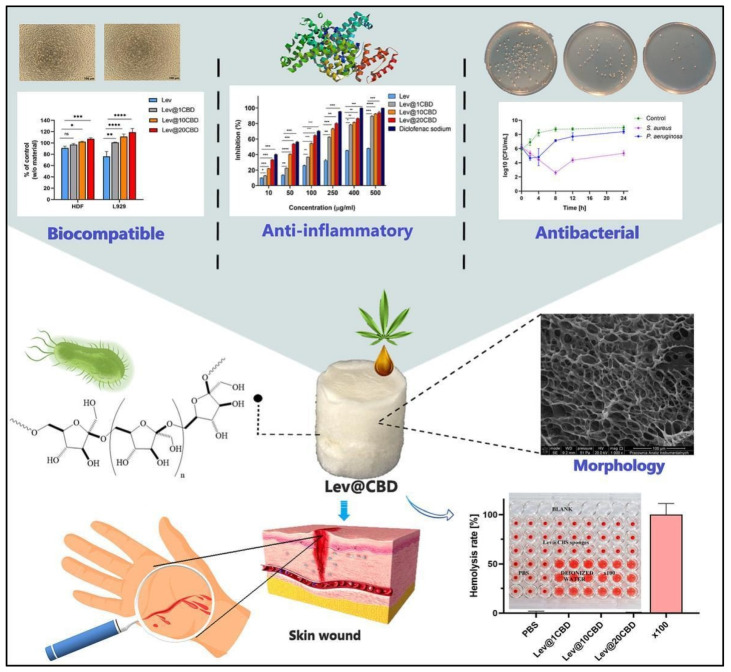
Novel, porous, antibacterial, levan-based sponges fortified with cannabis oil have demonstrated improved biocompatibility with L929 mouse fibroblasts and human dermal fibroblasts cells, enhanced anti-inflammatory potency, and antibacterial activity against *Staphylococcus aureus* and *Pseudomonas aeruginosa*. * *p* < 0.05, ** *p* < 0.01, *** *p* < 0.001, ns = non significant. These properties suggest excellent wound-healing efficacy. These pictures are reproduced with permission from [117] under a Creative Commons Attribution (CC-BY) license.

Similarly, in 2023, Chelminiak-Dudkiewicz and his team developed porous, antibacterial, levan-based sponges that incorporated cannabis oil [117]. These sponges exhibited favorable characteristics, including an optimal swelling ratio, water vapor transmission rate, and thermal stability and desirable mechanical and antioxidant properties. The sponges showed strong binding with fibrinogen and exhibited antibacterial activity against *Staphylococcus aureus* and *Pseudomonas aeruginosa*, while remaining non-hemolytic. They also demonstrated biocompatibility with L929 mouse fibroblasts and human dermal fibroblasts. Notably, the sponges maintained their non-hemolytic, anti-inflammatory, and antimicrobial properties, even after extended storage. These promising physicochemical and biocompatibility attributes suggest that these levan-based sponges could be a valuable addition to wound dressing technologies [117] (Figure 2).

**Figure 3:**
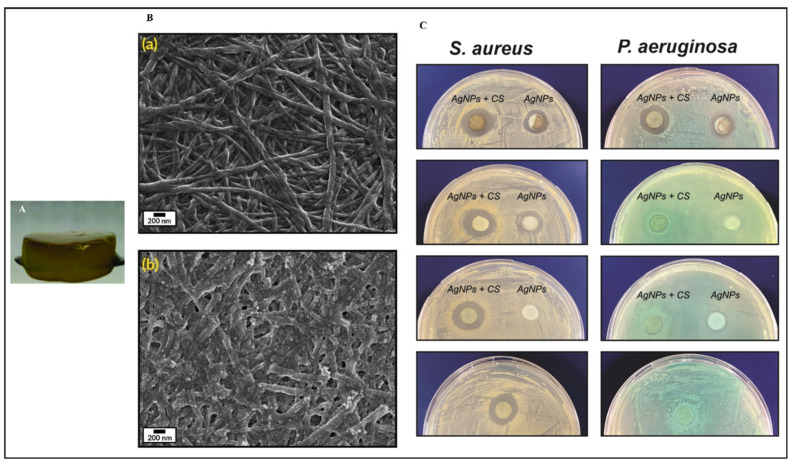
Collagen-based hydrogel fortified with silver nanoparticles and *Cannabis sativa* oil (**A**); scanning electron microscopy images of collagen hydrogel (**Ba**); and silver nanoparticles with *C. sativa* oil incorporated into the collagen hydrogel (**Bb**); as well as the antimicrobial efficacy of these hydrogels at varying concentrations against *Staphylococcus aureus* and *Pseudomonas aeruginosa* compared to control collagen (**C**). These pictures are reproduced with permission from [118] under Creative Commons attribution (CC-BY−4.0) license.

**Figure 4:**
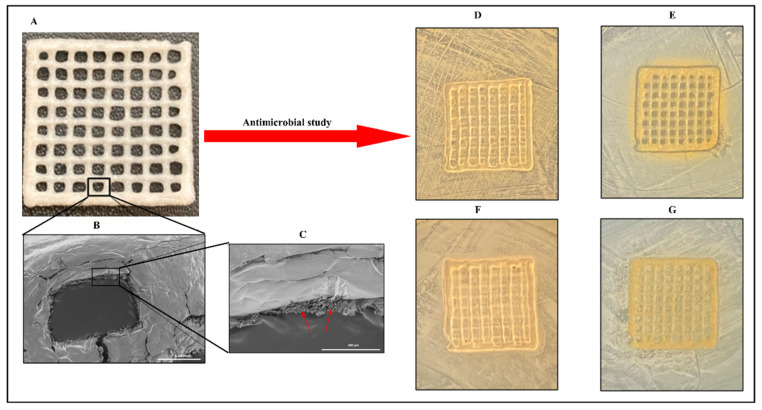
A three-dimensional, printed gelatin–alginate scaffold fortified with *C. sativa* oil. Lyophilized 3D-printed scaffold (**A**); scanning electron microscopy of 3D-printed scaffold (**B**); a single pore of the 3D-printed scaffold imaged through scanning electron microscopy (**C**); and the antimicrobial efficacy of a lyophilized gelatin–alginate scaffold fortified with or without *C. sativa* oil against *Staphylococcus aureus* (**D**,**F**) and *Escherichia coli* (**E**,**G**). Arrow marks indicate the porous structure in the freeze-dried composite. These pictures are reproduced with permission from [119] under a Creative Commons attribution (CC-BY−4.0) license.

A study describes the preparation of collagen hydrogels incorporating silver nanoparticles and *C. sativa* oil extract [118]. Collagen scaffolds loaded with 67 ± 7 mg/g of silver nanoparticles (AgNPs) were created by cross-linking collagen with an aqueous suspension of AgNPs. This hybrid biomaterial demonstrated sustained antimicrobial activity against both Gram-positive and Gram-negative bacteria for up to seven days. It also showed enhanced mechanical properties and resistance to enzymatic degradation. To improve the biocompatibility, *C. sativa* oil was added, significantly reducing the scaffold’s cytotoxicity to eukaryotic cells. This novel nanocomposite biomaterial presents a promising option for treating wound infections and promoting wound healing by inhibiting harmful bacteria, supporting new tissue growth, and ensuring high biocompatibility (Figure 3). Meanwhile, Antezana and colleagues have pioneered the use of *C. sativa* extract oil in 3D-printed gelatin–alginate (GEL-ALG) scaffolds, specifically designed for skin wound healing [119]. A GEL-ALG bioink with excellent printability was developed to create a biocompatible 3D scaffold infused with *C. sativa* oil extract. This scaffold provides both antimicrobial and antioxidant properties, making it a promising option for treating wound infections and enhancing wound healing (Figure 4). In a separate study, Lajoie et al. explored the effects of different emulsifiers including whey protein isolate, soy protein isolate, and Tween 80 on the encapsulation of cannabis oil using maltodextrin as a wall material [120]. The study evaluated the physicochemical properties of cannabis oil powders and their stability during in vitro digestion, focusing on bio-accessibility. The average diameters of fat globules in liquid nano-emulsions were 170 nm for the whey protein isolate (WPI), 259 nm for the soy protein isolate (SPI), and 95 nm for Tween 80 (Tw). The protein-based emulsifiers demonstrated high encapsulation efficiencies (>95%), whereas Tw showed a lower efficiency (~16%). The WPI-emulsified fat droplets remained stable at 176 nm when reconstituted in water, while the SPI and Tw droplets were larger at 346 nm and 210 nm, respectively. All the powders had high solubility (>97%). The peroxide value (PV) increased fourfold from the initial 5.2 mEq in the bulk oil upon extraction from the powder. An analysis of major cannabinoids—CBD, THC, and CBN—revealed no significant differences among the formulations compared to bulk oil, except for lower levels in Tw. In an in vitro digestion model, Tw increased cannabinoid bio-accessibility twofold (~53%) compared to the protein-based emulsifiers. This highlights the critical role of emulsifier choice in both encapsulation efficiency and cannabinoid bio-accessibility, underscoring the need for suitable emulsifying agents for the optimal oral delivery of cannabis oil.

**Figure 5:**
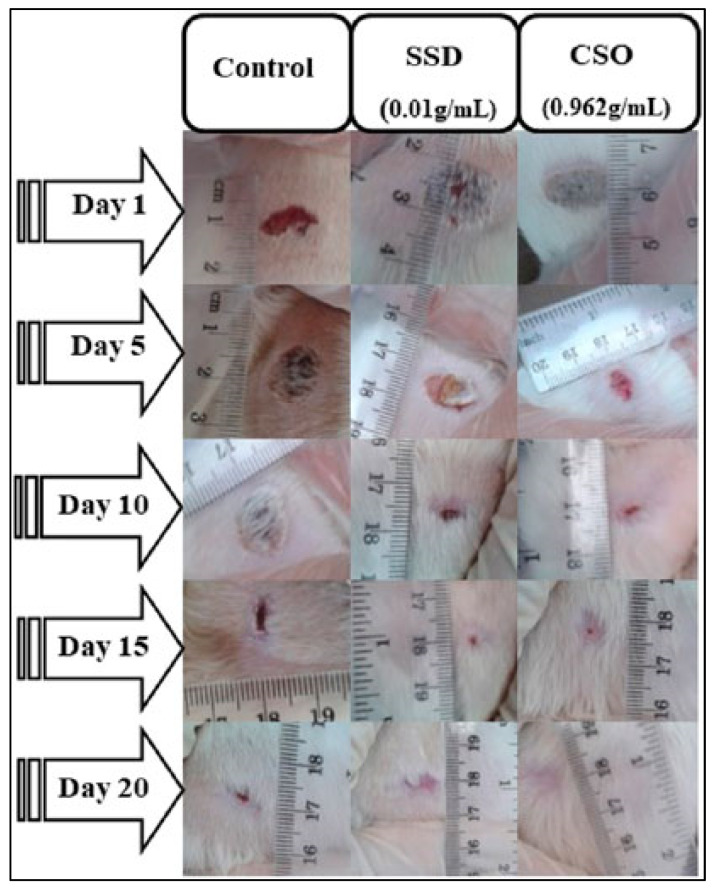
Effect of *Cannabis sativa* oil (CSO) on healing of burned skin in rats. The macroscopic morphology of burned skin wounds treated with cannabis sativa oil at 0.962 g/mL) and silver sulfadiazine (SSD) at 0.01 g/mL after 20 days demonstrates the promising effects of CSO in wound healing, showing comparable results to the standard medical treatment. Reproduced with permission from [121] under a Creative Commons attribution (CC-BY−4.0) license.

A study conducted by Bouarfa and colleagues investigates the wound-healing potential of cannabis oil and its anti-inflammatory properties using an animal model. The study aims to assess the effectiveness of cannabis oil in promoting wound recovery and reducing inflammation, providing insights into its potential therapeutic benefits in wound management [121]. In this study, Bouarfa and colleagues investigated the wound-healing potential of cannabis oil using 18 male albino Wistar rats divided into three groups: an untreated control, silver sulfadiazine-treated, and cannabis oil-treated. All the wounds started with a diameter of 1 cm. By the 5th day, both the cannabis oil and silver sulfadiazine treatments significantly reduced the wound size compared to the untreated control, with contraction rates of 53.95% for cannabis oil and 45.94% for silver sulfadiazine (*p* < 0.05) [121]. By the 15th day, the wounds in both the cannabis oil and the silver sulfadiazine groups were nearly healed, showing contractions of 98.8% and 98.15%, respectively. On the 20th day, the wounds treated with cannabis oil achieved complete healing (100%), while the silver sulfadiazine-treated wounds showed a 98.97% contraction. A histological analysis of the cannabis oil-treated wounds revealed granulation tissue, new blood vessels, fibroblasts, and collagen fibers. An in silico analysis identified arachidic acid, γ-linolenic acid, and linolenic acid as potent inhibitors of COX−1 and COX−2. Serum biochemical tests showed no significant changes in liver and kidney function in the cannabis oil-treated rats, whereas the silver sulfadiazine-treated rats had a significant increase in ALAT levels (*p* < 0.01). The study suggests that cannabis oil significantly promotes wound healing and tissue regeneration without adverse effects on liver or kidney function [121]. However, further research is needed to explore the molecular mechanisms underlying cannabis oil’s effects. Figure 5 illustrates these findings.

**Figure 6:**
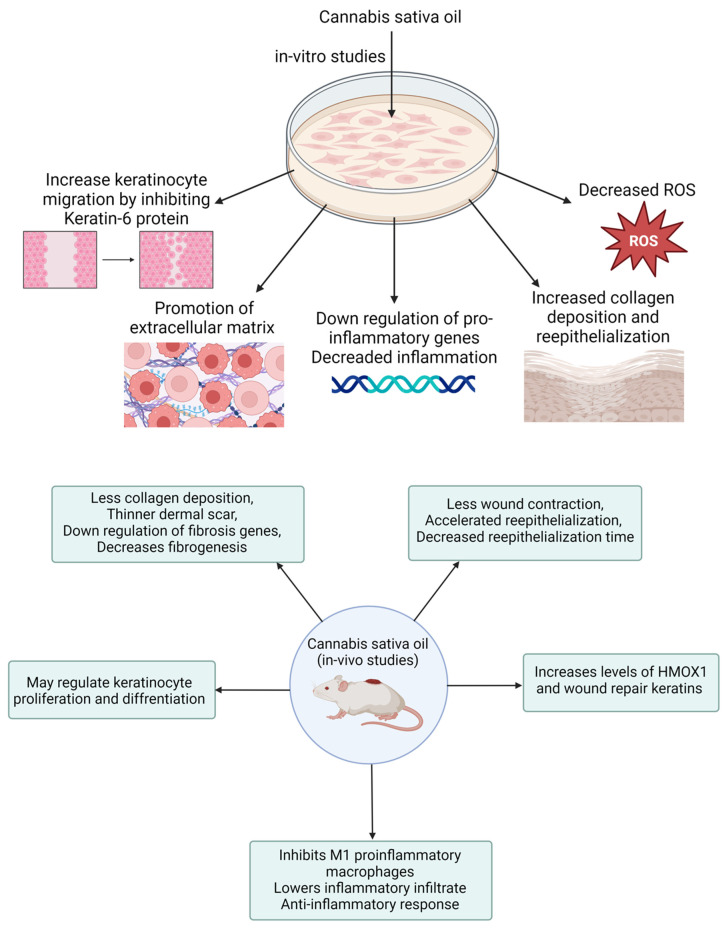
Schematic presentation of in vitro and in vivo performance processes of wound healing medicament.

In a related study, Zheng and colleagues developed a CBD-fortified, alginate-based hydrogel crosslinked with Zn^2+^ ions to enhance wound healing. The in vivo study showed that this hydrogel improved wound healing through a controlled inflammatory response, increased collagen deposition, enhanced granulation tissue formation, and enhanced blood vessel development. These effects were attributed to CBD’s properties, including its antioxidant, antibacterial, anti-inflammatory, and angiogenic activities [122]. Several case reports indicate that cannabis oil has been effective in treating wounds associated with conditions like epidermolysis bullosa and chronic pressure injuries. These reports highlight that cannabis oil use led to faster wound healing, reduced blistering, and alleviated pain, particularly when combined with cannabidiol [123,124]. These results underscore the necessity of conducting clinical trials to validate the effectiveness of cannabis oil in wound healing and management. Table 2 provides a summary of various preclinical studies investigating the role of cannabis and its derivatives in wound healing. Figure 6 demonstrates the in vitro and in vivo performance processes with their measurements.

## 6. Clinical Trials on Cannabis Oil/Extracts for the Pain Management

Despite the growing interest in CBD for various medical conditions, clinical trials specifically investigating its use in wound healing remain limited. However, numerous studies on the pharmacological, pharmacokinetic, and toxicological aspects of CBD provide valuable insights that could inform future research in this area. For example, NCT04841993 was a Phase 1 trial that explored the pharmacokinetics and pharmacological effects of different cannabis preparations, including a cannabis decoction, an oil, and a vapor. This study provided essential data on how these preparations interact with the body, offering a foundation for potential therapeutic applications. Another Phase 1 study, NCT04601207, investigated the bioavailability of CBD and THC from an emulsion product in healthy participants. The study aimed to understand how well these compounds are absorbed and utilized by the body, which is crucial for their clinical use. Another trial investigated the effect of Cybis™ 10:25 THC:CBD oil on patients with chronic refractory back and neck pain. This Phase 1/2 study explored the therapeutic potential of CBD and THC for treating chronic pain disorders, offering insights applicable to wound pain management. NCT04601207 was a randomized, triple-blind, comparator-controlled parallel study investigating the pharmacokinetics of cannabidiol and tetrahydrocannabinol in a novel delivery system, solutech, in association with cannabis use history, that demonstrated that the consumption of a test substance enhanced the pharmacokinetic parameters such as bioavailability, safety, and efficacy [140].

Despite extensive research into the pharmacological and pharmacokinetic properties of CBD, there are surprisingly few clinical trials focused specifically on its application in wound healing. However, these studies lay a strong foundation for understanding how CBD behaves in the body, its safety profile, and its potential therapeutic effects across various conditions. This underscores the need for targeted clinical research to explore the efficacy of CBD in wound healing. Such research could bridge a significant gap in medical knowledge and pave the way for developing effective new treatments for wound care.

## 7. Conclusions

Cannabis oil, especially its primary bioactive constituents, CBD and THC, demonstrates considerable potential in facilitating skin wound healing by modifying oxidative stress via the regulation of reactive oxygen species. CBD’s therapeutic effects in wound healing are largely attributed to its antioxidant, anti-inflammatory, and antimicrobial properties. Increased ROS levels can hinder wound healing by exacerbating inflammation and cellular damage; however, CBD’s antioxidant properties mitigate these effects, fostering a more conducive environment for tissue regeneration. Additionally, the antibacterial and analgesic properties of cannabis contribute to reducing the microbial load and minimizing the complications associated with chronic wounds, thereby enhancing the overall healing efficacy. Integrating cannabis oil into drug delivery systems for wound management represents a promising strategy for treating both acute and chronic wounds. However, further clinical research is needed to fully understand the mechanisms involved and to optimize the therapeutic applications of cannabis oil in wound care.

## Figures and Tables

**Table 1:** Summarized chemical constituents present in the *C. sativa* (L.) plant and their activities.

Terpenes from Hemp Oil	Role	References
α-Humulene	Promotes anticancer activity. Prevents hepatocellular carcinoma through the inhibition of the release of Akt and inhibits its proliferation. Promotes anti-inflammatory activity through the inhibition of NF-κB.	[106,107]
β-caryophyllene	Promotes antitumor activity against breast cancer cells. Promotes the synergistically cytotoxic effect of doxorubicin. Attenuates the increase in malondialdehyde levels in the kidney. Preserves the tissue metabolism of vessels, hindering the results from a bilateral common carotid artery occlusion followed by reperfusion. Neuroprotective effect. Promotes anti-inflammatory and antioxidant activities. Promotes antinociceptive action.	[107,108,109,110,111]
β -caryophyllene oxide	Promotes antineoplastic activity. Prevents cancer through the alteration of cancer pathways.	[112]
α-pinene and β-pinene	Inhibits the activity of acetylcholinesterase in the brain. Possesses antiseptic properties.	[113]
α-pinene	Antibacterial, anti-inflammatory, bronchodilator, and gastroprotective activities.	[114]
β-myrcene	Promotes the analgesic effect of THC and CBD.	[115]

**Table 2:** Preclinical studies that assess the application of CBD and its various preparations in wound healing.

Agent	Findings	References
**In vitro studies**		
CBD, phytosterols, and unsaturated fatty acids extract	Stimulate cell migration and proliferation and enhance MMP activity. Reduce cytokine expression in a CBD-dependent manner. Influence ECM-related gene expression. Activate skin cell matrix remodeling in a CBD and β-sitosterol-dependent manner.	[125]
*C. sativa* L. extract and CBD	Inhibit the release of inflammation mediators. Counteract TNF-a-induced NF-kB signaling. Downregulate genes involved in skin inflammation.	[126]
*C. sativa* L. herb extract	Inhibit the MMP, collagenase, and elastase activity. Unaffected trans-epidermal water loss and skin hydration. Reduce oxidative stress, inhibit skin aging, and improve skin cell viability and hydration.	[127]
CBD and cannabigerol nanoparticles	Nanoparticles exhibited regulated release profiles, improved thermal stability of the entrapped components, and high entrapment efficiency. Cannabigerol nanoparticle was safe up to 48 h. In vitro wound healing showed early promotion, followed by inhibitive effects.	[128]
CBD nano-emulsion	Nano-formulated CBD exhibited lower cytotoxicity and higher anti-inflammatory activity. Neither the nano-emulsion nor pure CBD penetrated the cornea after 4 h of treatment. Treatment with nano-emulsion showed that 94% of the initial CBD remained in the apical compartment, while only 54% of pure CBD was measured.	[129]
Nano-emulsion of hemp essential oil extract	An equivalent cytotoxicity between EOs and NEs showed lower expressions of the pro-inflammatory cytokines. The basal level of the inflammatory cytokines remained unaltered.	[130]
Fibroin film incorporating CBD/2-Hydroxypropyl--cyclodextrin	CBD/HP-β-CD complex with a 1:2 molar ratio provided an 81.5% *w*/*w* content of CBD. XRD and FTIR demonstrated the successful encapsulation of CBD into the HP-β-CD cavity. Human dermal fibroblasts showed cell proliferation to increase, migration enhanced in a scratch assay, and VEGF protein expression increased.	[131]
Semi solid hydrogels loaded with CBD/β-Cyclodextrin	Hydrogels with different CBD/β-CD contents demonstrated good release profiles and antioxidant properties with antibacterial activity. No cytotoxicity below a concentration of 1.25 mg/mL was observed. Nitric oxide production was inhibited by over 75%.	[132]
Cannabis sativa (Hemp) seed-derived peptides (WVYY and PSLPA)	Antioxidants, antibacterial agents, and wound-healing promoters demonstrated by peptides derived from hemp seed. Antioxidants and anti-inflammation effect depended essentially on the Nrf2 signaling pathway.	[133]
**In vivo studies**		
A combination of oils from different plants	NF treatment significantly improved wound contraction. NF treated group showed a significantly faster epithelialization time.	[134]
CBD	Inflammatory scores in the CBD-treated lesions were significantly lowered. CBD has indicated an early anti-inflammatory effect.	[135]
Topical 1% CBD	Insignificant difference in healing process of wound, compared among tested topical.	[136]
*C. sativa* L. essential oil	Essential oil demonstrated anti-inflammatory activity. Reduced level of neutrophils in blood and skin tissue with promoted wound healing. The oil reversed the incision-induced neurobehavioral changes.	[137]
CBD	The deletion of cannabinoid receptors CB2 escalate interleukin (IL)−6 and TNF-α however, the tissue regeneration was unaffected. While deletion of cannabinoid receptors CB1 wound closure was delayed in early phases of healing, which ultimately improved the concentration of monocyte chemoattractant protein, tumor necrosis factor.	[138]
*C. sativa* L. seed@graphene quantum dots	*C. sativa* L. seed@graphene quantum dots demonstrated improved antibacterial activity. *C. sativa* L. seed@graphene quantum dots demonstrated improved anti-inflammatory activity. An accelerated re-epithelization and granulation tissue formation.	[139]

## Data Availability

Data can be made available on request to corresponding authors.

## Abbreviations

Reactive oxygen species (ROS); oxidative stress (OS); superoxide anion (O^2•−^); hydrogen peroxide (H_2_O_2_); hydroxyl radical (^•^OH); reduction–oxidation (redox); cannabidiol (CBD); tetrahydrocannabinol (THC); ultraviolet B (UV-B); vascular endothelial growth factor (VEGF); nitric oxide (NO); phosphoinositide−3 kinase (PI3 K); basic fibroblast growth factor (bFGF); c-Jun N-terminal kinases (JNK 1/2); extracellular regulated signaling kinase (ERK 1/2); kinase insert domain receptor (Flk−1/KDR genes); transforming growth factor beta (TGF-β); matrix metalloproteinases (MMPs); tissue inhibitors of metalloproteinases (TIMPs); mitogen activated protein kinase (MAPK pathways); interleukin (IL); prostaglandin (PGE_2_); tumor necrosis factor alpha (TNF-a); nicotinamide adenine dinucleotide phosphate (NADPH); extracellular matrix (ECM); nuclear factor kappa-light-chain-enhancer of activated B cells (NF-κB); epidermal growth factor (EGF); epidermal growth factor receptor (EGFR); platelet-derived growth factor (PDGF); negative pressure wound therapy (NPWT); hyperbaric oxygen therapy (HBOT); mouse fibroblasts cells (L929); gelatin–alginate (GEL-ALG); soy protein isolate (SPI); Tween 80 (Tw); cyclooxygenase (COX); *Cannabis sativa* oil (CSO); silver sulfadiazine (SSD); 2-hydroxylpropyl-β-cyclodextrin (HP-β-CD); Proline–Serine–Leucine–Proline–Alanine (PSLPA); Tryptophan–Valine–Tyrosine–Tyrosine (WWVY); Cannabinoid Receptor 1 (CB1); Cannabinoid Receptor 2 (CB2).
